# Recurrence and Driving Factors of Visceral Leishmaniasis in Central China

**DOI:** 10.3390/ijerph18189535

**Published:** 2021-09-10

**Authors:** Yingze Zhao, Dong Jiang, Fangyu Ding, Mengmeng Hao, Qian Wang, Shuai Chen, Xiaolan Xie, Canjun Zheng, Tian Ma

**Affiliations:** 1 National Institute for Viral Disease Control and Prevention, Chinese Center for Disease Control and Prevention (China CDC), Beijing 102206, China; zhaoyz@ivdc.chinacdc.cn; 2 State Key Laboratory of Resources and Environmental Information System, Institute of Geographic Sciences and Natural Resources Research, Chinese Academy of Sciences, Beijing 100101, China; jiangd@igsnrr.ac.cn (D.J.); haomm@igsnrr.ac.cn (M.H.); chens.17s@igsnrr.ac.cn (S.C.); xiexl.20b@igsnrr.ac.cn (X.X.); 3 College of Resources and Environment, University of Chinese Academy of Sciences, Beijing 100049, China; 4 Department of Earth System Science, Tsinghua University, Beijing 100084, China; wangqth@mail.tsinghua.edu.cn; 5 Chinese Center for Disease Control and Prevention (China CDC), Beijing 102206, China; zhengcj@chinacdc.cn

**Keywords:** visceral leishmaniasis, spatiotemporal distribution, recurrence, driving factors

## Abstract

Visceral leishmaniasis (VL) is an important vector-borne zoonosis caused by *Leishmania* spp. that has been spreading in China. It has been posing a significant risk to public health in central China due to its recurrence in recent decades. Yet, the spatiotemporal patterns and the driving factors of VL in central China remain unclear at present. The purpose of this study was to analyse spatiotemporal distribution, explore driving factors, and provide novel insight into prevention and control countermeasures of the VL spreading in central China. Based on data of human VL cases from 2006 to 2019 obtained from the Chinese Centres for Disease Control and Prevention (CDC), we depicted the map showing the spatiotemporal distribution of VL in central China. We further explored the driving factors contributing to the spread of VL through the general additive model (GAM) by combining maps of environmental, meteorological, and socioeconomic correlates. Most VL cases were reported in Shaanxi and Shanxi provinces, the number of which has been increasing every year in the last 14 years, from 3 new cases in 2006 to 101 new cases in 2019. The results of GAM revealed that environmental (i.e., changes in grasslands/forests), meteorological (i.e., temperature and relative humidity), and socioeconomic (i.e., population density) factors are significantly associated with the prevalence of VL in central China. Our results provide a better understanding regarding the current situation and the driving factors of VL in central China, assisting in developing the disease prevention and control strategies implemented by public health authorities.

## 1. Introduction

Visceral leishmaniasis (VL) is an important parasitic zoonotic disease caused by *Leishmania* spp. and transmitted by infected female phlebotomine sand flies (i.e., *Phlebotomus* *chinensis* and *P. longiductus*), the hosts of which include animals such as canids, rodents, marsupials, hyraxes, and human beings [1,2,3,4]. VL generally affects the spleen, liver, and other lymphoid tissues. The main clinical manifestations of this disease are usually chronic irregular fever, splenomegaly, anaemia, emaciation, leukopenia, and increased serum globulin, which can be fatal without treatment [2,5]. As one of the deadliest parasitic diseases in the world, VL causes nearly 500,000 new cases globally each year, of which approximately 60,000 end up dead, second only to malaria in numbers of fatalities [6,7,8], afflicting millions of people worldwide. For receiving inadequate public attention and having high mortality rates in the world, especially in poverty-stricken regions, the World Health Organisation (WHO) has declared VL a neglected tropical disease (NTD) [6].

Recently, there has been an increasing interest in the study of factors driving VL, presented in some previous studies of factors that contribute to the emergence, spread, and endemicity of this disease. For example, in recent years, there has been evidence suggesting that the disease-transmitting sand fly population varies greatly depending on the landscape, more specifically, elevation, and land cover [9,10]. Additionally, previous research has established that precipitation and humidity could influence the breeding and harbouring of sandflies [11,12], while temperature affects both the development of the infecting *Leishmania* parasite in the sandfly and the life cycle of the vectors [13]. Aside from exploring desirable environments for the survival of the disease transmitters, Reis et al. have revealed in their research a negative correlation between comparably high altitudes and the prevalence rate of VL in a few municipalities [11]. In addition, VL, as a neglected tropical disease, is strongly associated with socioeconomic factors. The situation in which environmental sanitation resulting from poor housing conditions coupled with the lack of personal protective measures will prompt a surging exposure of humans to infected sand flies [14].

VL is one of the severest parasitic diseases that China has fought for a long time [15]. In the 1950s, VL was prevalent in vast rural regions located in the north of the Yangtze River, affecting nearly 665 counties scattered in 16 provinces, municipalities, and autonomous regions (Shandong, Jiangsu, Anhui, Henan, Hebei, Shaanxi, Gansu, Xinjiang, Qinghai, Ningxia, Sichuan, Hubei, Shanxi, Liaoning, Inner Mongolia, and Beijing), with the prevalence rate ranging from 10/100,000 to 500/100,000 in each county [16]. There were estimated 530,000 VL cases occurred in China, causing mass mortality, serious issues of public health, and national security [17]. After 1950, all endemic provinces and regions had established professional institutions for the prevention and control of VL under the guidance of the central government [18]. Control measures such as the diagnosis and treatment of patients, health education, dog management and control (i.e., identification, isolation, and disposal of infected dogs), and sandfly control (i.e., residential insecticide indoor spraying) were implemented [15]. With these control measures conducted by the national and local governments, VL was tightly controlled in China, and the eastern part of the country almost achieved the elimination of VL in the 1960s [17,19]. However, through the implementation of the national programme for developing western and northwestern China since the late 1980s, the increasing habitats suitable for VL transmission have led to the re-emergence of VL in the areas where VL was once epidemic [19]. In the 1990s, 2629 new VL cases were reported, and the number of infected counties gradually increased to 43, distributed mainly in Xinjiang, Gansu, Inner Mongolia, and Sichuan [17,18]. During the subsequent years from 2004 to 2016, VL was reported in 83 counties of 7 provinces and autonomous regions (Xinjiang, Gansu, Sichuan, Henan, Hebei, Shaanxi, Shanxi), with a total number of 3337 new VL cases [20].

The number of recurrent VL cases in central China has been on the rise in recent years [20,21]. During the period from 2006 to 2019, the number of annual new VL cases officially reported in central China (Shanxi, Shaanxi, Henan, and Hebei) increased from 3 to 101, and the number of reported counties increased from 3 to 33, posing a growing public health burden on the local government. Yet, with the recorded cases from past decades, the spatiotemporal distribution patterns and the ways these driving factors affect the prevalence of VL in central China remain unclarified at present. Thus, based on reported human VL cases from 2006 to 2019, we aim to analyse the spatiotemporal distribution and explore the driving factors of VL to eventually provide novel insight into disease prevention and control countermeasures to the spread of VL in central China.

## 2. Materials and Methods

### 2.1. Study Area

**Figure 1:**
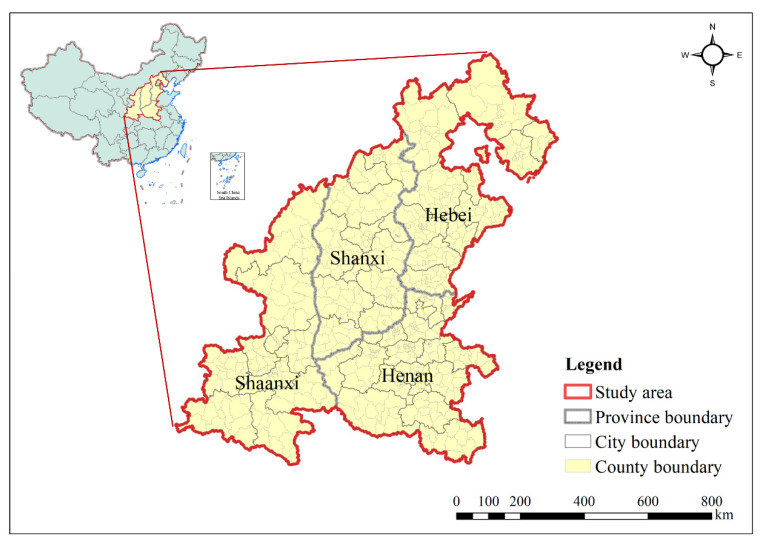
The study area adopted in this study. Central China included provinces of Shaanxi (city: 11, county/district: 108, population: 38.76 million, area: 0.20 million km^2^), Shanxi (city: 11, county/district: 119, population: 37.29 million, area: 0.16 million km^2^), Henan (city: 18, county/district: 169, population: 96.4 million, area: 0.17 million km^2^), and Hebei (city: 11, county/district: 176, population: 75.92 million, area: 0.19 million km^2^).

In the present study, Shanxi, Shaanxi, Henan, and Hebei provinces were determined as the study area located in central China, stretching from 105°29′ E to 119°50′ E, 31°42′ N to 42°40′ N, with a total area of 0.72 million square kilometres and a permanent population of 248.4 million (Figure 1). The climate in the study area is temperate, with an average temperature of 10–15 °C and average annual precipitation of 500–600 mm. The altitude of the study area ranges from −4 to 3753 m, with an overall characteristic of being higher in the western regions (Shaanxi and Shanxi) than in the eastern parts (Henan and Hebei). Among these provinces, Shanxi and Shaanxi are mainly mountainous plateaus, while Henan and Hebei are mainly plains.

### 2.2. Data Collection

#### 2.2.1. Human Visceral Leishmaniasis

In the present study, data of human VL cases spanning from 2006 to 2019 were obtained from the Chinese Centres for Disease Control and Prevention (CDC) (http://www.chinacdc.cn, Access date: 10 November 2020), among which clinically diagnosed and laboratory-confirmed cases were selected as confirmed human VL cases, resulting in a total of 366 cases. On this basis, we calculated the annual VL totals of each county in the study area from 2006 to 2019 according to the unique county code. R version 3.5.2 (https://www.r-project.org/, Access date: 1 September 2019) was employed for data processing. All data used in the present study were de-identified to protect patient confidentiality.

#### 2.2.2. Driving Factors

The prevalence of VL has been proven to be influenced by multiple factors, falling into three broad categories: environmental, meteorological, and socioeconomic factors [22]. Changes in these factors are likely to lead to fluctuation in the prevalence and distribution of the disease by affecting the habitat of vectors (phlebotomine sand flies) and animal hosts, as well as the interaction of humans and vectors or animal hosts [23]. Detailed information on the possible driving factors adopted in this study is listed in Table 1.

#### Environmental Factors

Several studies have pointed out that terrain has a significant influence on the presence of VL [9,24,25,26]. For example, a study conducted by Jiang et al. claimed elevation as the most important predictor that is strongly correlated with VL in western and central China based on analysis through a boosted regression model [9]. In this study, elevation was assumed to be a potential driving variable for VL prevalence. The elevation dataset with a spatial resolution of 90 m generated by the SRTM was downloaded from the CGIAR Consortium for Spatial Information (http://srtm.csi.cgiar.org, Access date: 15 March 2020).

Land cover, especially vegetation (such as forest and grass), has been frequently considered to influence populations of vectors and their interaction with susceptible persons [10], thus affecting the occurrence of human VL cases. Prior studies have noted that the increase in vegetation cover has been associated with the rise in the prevalence of human VL [24,27]. On the other hand, evidence suggests that changes such as deforestation related to physical characteristics of the landscape or biodiversity loss itself could favour disease-carrying hosts or vectors, as well as increasing the efficacy of disease transmission to remaining hosts [28]. The fact that the changes in forest and grass can, to some extent, represent good proxy measures for quantifying vegetation change leads us to assume them to be two key variables for the prevalence of VL in the present study. The annual forest and grassland gridded data with a spatial resolution of 5.5 km spanning from 2006 to 2019 were generated by ArcGIS 10.2 software (ESRI, RedLands, CA, USA). The origin data adopted in this study were the Level-3 MODIS product—The Land Cover Type Yearly Climate Modelling Grid (CMG, namely, MCDI2C1), providing the dominant land cover type and the sub-grid frequency distribution of land cover classes, available on the website of the Atmosphere Archive and Distribution System of NASA (https://ladsweb.modaps.eosdis.nasa.gov/, Access date: 3 February 2021); Further detailed information can be found elsewhere [29].

#### Meteorological Factors

Previous studies have noted the importance of meteorological factors on the habitat of the vector, as well as the size and longevity of its population [12,13], where strong relationships between meteorological factors and VL have been reported constantly [10,12,25,30,31]. For instance, a study conducted by Gao et al. established a connection between precipitation, relative humidity, and the VL spread in the Xinjiang Uygur Autonomous Region of China [25]. Similar results reported in Brazil also found that precipitation correlates with the prevalence rate of VL positively [11]. In this study, precipitation, temperature, and relative humidity, as important explanatory variables, were implicated in the spatial–temporal distribution of VL. ANUSPLIN-SPLINA software used in the previous literature [9,32,33] was employed to generate gridded datasets of annual meteorological datasets with a spatial resolution of 1 km. The original meteorological data were downloaded from the China Meteorological Data Service Centre (http://data.cma.cn, Access date: 1 November 2019) by monitoring stations on a daily basis.

#### Socioeconomic Factors

Previous studies illustrated that socioeconomic factors such as population density, gross domestic product (GDP), urbanisation, migration, etc. were associated with human VL presence [14,30,34]. For example, a review demonstrated that the prevalence of VL was positively correlated with population and urbanisation in Brazil [22]. Therefore, for factors representing socioeconomics in this study, we adopted the data of population and GDP (a proxy for the regional development level), which were obtained from the Socioeconomic Data and Applications Centre (SEDAC) (https://sedac.ciesin.columbia.edu, Access date: 16 May 2020) and the website of the Global Change Research Data Publishing and Repository (http://www.geodoi.ac.cn, Access date: 16 May 2020), respectively.

Human movement is a key behavioural factor in many vector-borne disease systems because of its influence on human exposure to vectors and thus affects the transmission of pathogens [30]. Some studies also revealed that the entry of a nonimmune person into an epidemic area with existing vector-borne diseases may lead to new infection cases [35]. Moreover, human movement has been used to predict the transmission of several vector-borne diseases (i.e., Dengue and Zika fevers) [36,37] and their vectors (i.e., *Aedes* mosquitoes) [38,39]. In this study, we adopted an urban accessibility dataset as a proxy indicator for patterns of human movement. The approximately 1 km × 1 km gridded urban accessibility dataset was downloaded from the European Commission Joint Research Centre (http://forobs.jrc.ec.europa.eu/, Access date: 24 April 2020).

### 2.3. Modelling Approach

The general additive model (GAM), as a common statistical model, has been widely employed in discovering the links between diseases and environmental factors, which is useful to elucidate nonlinear statistical relationships [40,41,42]. In our study, GAM with a Poisson distribution (Equation (1)) was used to explore the associations between various spatial predictors (environmental, meteorological, and socioeconomic) and the number of VL cases in central China from 2006 to 2019.

(1) $$\begin{array}{l} Y_{i,t}=f(X_{ele(i,t)})+f(X_{cf(i,t)})+f(X_{cg(i,t)})+f(X_{pre(i,t)})+f(X_{tem(i,t)})+f(X_{rh(i,t)})\\ +X_{pd(i,t)}+X_{gdp(i,t)}+X_{ua(i,t)}+Endemic_{i}+\varepsilon \end{array}$$

where *i* = county, *t* = time (year), Y*_it_* means the number of VL cases in county*_i_* in year*_t_*, *f* means a smoothing function. Specifically, *X_ele_* expresses the average altitude (m) of county*_i_*, and *X_cf_* and *X_cg_* refer to changes in the forest and grassland cover in county*_i_* and in year*_t_*, respectively. *X_tem_*, *X_rh_*, and *X_pre_* indicate averaged mean temperature (°C), relative humidity (%), and precipitation (mm) in county*_i and_* in year*_t_*, respectively. *X_pd_* means the mean population density (the number of people per square km) of county*_i_*, *X_gdp_* represents the mean value of GDP in county*_i_*, *X_ua_* refers to mean urban accessibility (specifically, the estimates of the travel time to a city of 50,000 people or more) of county*_i_*. In light of the different impacts of spatial predictors on endemic or nonendemic regions in central China, the term ‘*endemic’* was designed as the fixed effect term, which represents whether the county*_i_* is endemic to VL or not. In the present study, ‘endemic county’ can be defined as follows: ‘a county with reported local VL cases in the past 10 years’. Detailed information about endemic counties in central China was listed in Table 2. Additionally, ε is the error term.

We employed version 3.5.2 (the 64 bit version) of R to build the GAM model and implemented our analyses using the extension package ‘mgcv’ in R. There were two essential parts in the GAM modelling process: (a) a comprehensive dataset of human VL cases at the county level and (b) a suite of datasets on spatial covariates of VL. In this study, all spatial data were transformed into the same geographic coordinate system (WGS-84), and the same projected coordinate system (Albers conical equal area). After that, the gridded spatial predictors were transformed at the county level based on ArcGIS 10.2 and Python 2.7. Finally, we spatially matched the confirmed VL cases with multiple spatial covariates according to the unique county codes.

## 3. Results

### 3.1. Spatiotemporal Distribution Characteristics

**Figure 2:**
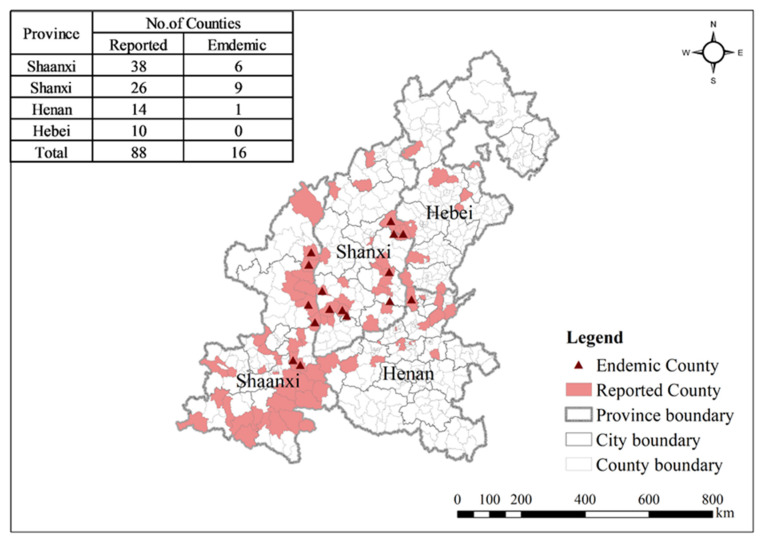
The spatial distribution of VL in central China from 2006 to 2019.

Figure 2 depicts the spatial distribution of VL in central China where the red areas were reported having VL cases from 2006 to 2019. The red triangle symbols represent counties where VL was historically endemic with reported local VL cases in the past 10 years [20,43]. From Figure 2, we found 88 counties that were reported with VL cases in central China from 2006 to 2019. The disease was distributed mainly in the eastern, southern, and central parts of Shaanxi Province, as well as the southwestern and eastern parts of Shanxi Province. Other scattered VL cases occurred in the northern part of Shanxi and Henan and central Hebei. Among the 88 case reporting counties, 72.7% (64/88) were concentrated in Shaanxi and Shanxi provinces, of which Shaanxi accounted for 43.2% (38/88) while Shanxi accounted for 29.5% (26/88). Compared with Shanxi and Henan provinces, fewer counties (24) were reported with VL cases in Hebei and Henan provinces. According to previous studies, 16 counties listed in Table 2 were defined as VL endemic counties in central China, distributed mainly at the junction of Shanxi and Shaanxi. Eastern and southeastern Shanxi contains the most endemic counties (nine endemic counties), followed by Shaanxi (six endemic counties) and Henan (one endemic county). No VL endemic county was found in Hebei.

Table 3 presents the detailed temporal distribution of VL cases in infected provinces in central China from 2006 to 2019 and the fact that the number of new VL cases increased annually during the study period. For instance, the total number of new cases has grown from 3 new cases in 2006 to 101 new cases in 2019. In addition, the number of VL cases has surged at a rate of nearly 1–2 times per year since 2016, with 29 new cases in 2016, 58 new cases in 2017, and 101 new cases in 2019. Overall, a total of 366 VL cases were reported in central China during this period, of which 88% (322/366) were reported in Shaanxi and Shanxi provinces, with 161 cases reported in Shaanxi and Shanxi provinces, respectively, while there were only 30 and 14 cases reported in Henan and Hebei.

**Figure 3:**
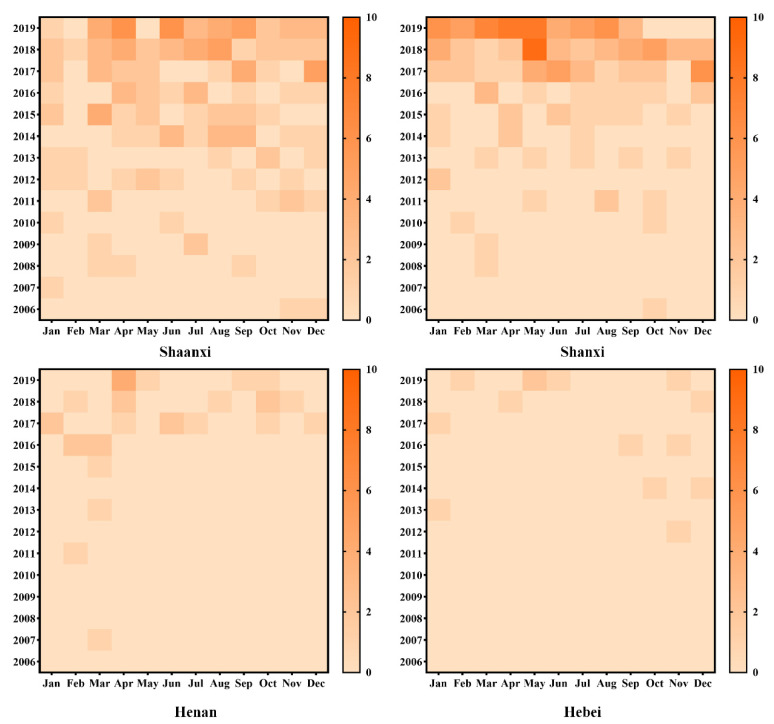
The interannual variability in the number of VL cases in central China (Shaanxi, Shanxi, Henan, and Hebei) from 2006 to 2019.

Figure 3 further shows the interannual variability from 2006 to 2019 of the number of reported VL cases in the study area, revealing an annually increasing trend in the number of VL cases in Shaanxi, Shanxi, Henan, and Hebei. Additionally, the numbers of each year’s new cases in Shaanxi and Shanxi were significantly greater than that in Hebei and Henan. In addition, we also found that the months when a large number of VL new cases show up in each province vary slightly among regions. For example, in Shaanxi Province, VL seems to occur more frequently in summer and autumn, while in Shanxi Province, it is more likely to occur in winter and spring.

### 3.2. Driving Factors of VL Recurrence

**Figure 4:**
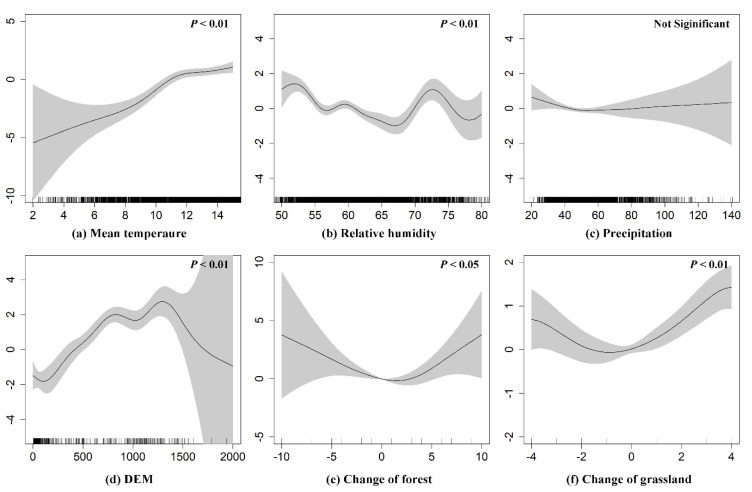
The relationships of spatial predictors on VL cases in central China: (**a**) mean temperature; (**b**) relative humidity; (**c**) precipitation; (**d**) DEM; (**e**) change in forest; (**f**) change in grassland.

The summary statistics based on the GAM model for spatial predictors are presented in Table 4, from which we could conclude that except for precipitation (*P* = 0.262), all the other variables were significantly associated with VL at the significance level of *P* < 0.1. Among these variables, elevation, change in grassland, mean temperature, relative humidity, and population had substantially significant associations with VL at the significance level of *P* < 0.001. Changes in forest and urban accessibility had a significant correlation with VL at the level of *P* < 0.05. Population was related to VL at the significance level of *P* < 0.1. Figure 4 depicts the relationships of risk factors to the prevalence of VL in central China, from which we could see that there was a significant positive correlation between mean temperature and VL. A complex nonlinear, yet generally negative, link occurred between the variables of relative humidity and VL. There was a significant positive correlation between elevation and VL when below 1400 m, while a negative correlation occurred when the altitude was higher than 1400 m. In addition, intriguingly, the relationships of changes in forest and grass to VL were revealed to be U-shaped curves in this study.

## 4. Discussion

Owing to the re-emergence of the disease in recent decades, VL remains a non-negligible threat to public health in central China. However, little is known about the VL distribution patterns, as well as what factors have been driving the recurrence of the disease in central China. For designing a future control policy, it is imperative to elucidate the current epidemiological characteristics and risk factors for VL. Thus, through analysing the spatiotemporal distribution of VL and identifying the driving factors related to the disease in central China during the period from 2006–2019, we attempt to provide novel insight into the prevention and control of VL.

In this study, we first depicted the spatial–temporal distribution of VL in central China from 2006 to 2019. The results indicated that VL was more prevalent in Shaanxi and Shanxi, compared with Henan and Hebei. A possible explanation would be the mountainous areas of Shaanxi and Shanxi provinces providing rather suitable living conditions for the vectors and canine hosts of VL. In addition, our study found that the number of VL cases has increased over time during the last 14 years, from 3 new cases in 2006 to 101 new cases in 2019. One reason for the continued growth in new cases could be the growing number of domestic dogs, accelerating population mobility, and changes in land use. On this basis, we explored the association of environmental, meteorological, and socioeconomic factors with VL based on the GAM model, providing insight into the prevention and control of VL in the future.

The results of this study indicate that environmental, meteorological, and socioeconomic factors significantly correlate with the prevalence of VL in central China. For meteorological factors, the current study found that there was a significant positive correlation between mean temperature (0–14 °C) and VL, which is in line with previous research, suggesting that the risk of VL increases with the rise in temperature [44]. The most likely cause of this result would be the sandfly metabolism that influences the oviposition, defecation, hatching, and adult emergence rates would increase with rising temperature [45]. However, another study suggests that the lifespan of the disease-carrying sandfly adults increases with decreasing temperature within a range of 18–32 °C [46]. A complex nonlinear, but generally negative, association was observed between relative humidity and VL, which is consistent with the results of analyses conducted by Li et al. and Ding et al. in the Xinjiang Uygur Autonomous Region, China [24,44]. In addition, the relationships between meteorological factors and VL have been proven to show divergent patterns in studies of different regions. For example, a study conducted by Reis et al. found that VL was positively correlated with precipitation while negatively with the average temperature in Brazil [11], differing radically from our results of this study in central China, which could be explained by geographic heterogeneity. For environmental factors, there was a significant positive correlation between elevation and VL at altitudes below 1400 m, possibly owing to the effect from the increase in altitude on the habitat (i.e., grassland and scrubland) distribution of disease vectors or rodent hosts [25], but a negative correlation occurred when above 1400 m. Similar findings have been reported in a previous study where a negative correlation was found between municipalities with higher altitudes and the prevalence rate of VL [11]. In our results, the most intriguing finding is that the relationships between the change of forest (grass) and VL were observed as a U-shaped curve, indicating the probability of people becoming infected with VL increases with the extent of changes in forest and grassland, whether it is positive or negative. Land-use change has been connected to infectious disease risk in previous studies. On the one hand, green areas have previously been observed to be positively associated with the occurrence of VL [24,27], given that the dense vegetation is favourable for vector populations and perhaps sylvatic canine reservoir hosts. On the other hand, the loss of green area (i.e., forest and grassland) is primarily caused by human activities such as deforestation or urbanisation that could change the habitat of vectors or animal hosts and thus increase the human exposure to vectors or animal hosts. Our results also suggest that dramatic changes in land use caused by human activities may increase the risk of disease transmission. In addition, the results of this study reveal a significant association between socioeconomic factors and VL, which is consistent with previous studies showing that socioeconomic factors are playing an increasingly important role in human infectious diseases because of their effect on housing, environmental sanitation, healthcare conditions, personal health level, and the frequency of human movement [14].

This study has some limitations that should be mentioned. On the one hand, vectors and dogs, as important parts of the whole transmission cycle of VL, have been demonstrated to have a geographical distribution consistent with sick dogs and vectors by many researchers [47]. However, this study was unable to encompass biological factors (dogs and vectors) owing to data constraints. On the other hand, individual factors (i.e., age, sex, and the inhabitants’ habits) were not included in this study, although the variations in them were considered important risk factors relating to VL prevalence in previous studies. For example, a study conducted in Shaanxi Province indicated that men, children, farmers, and dog owners were more likely to become infected with VL, compared with others, while no significant correlation was found between the frequency of using mosquito nets or mosquito coil and catching VL [48]. In future research, we will try to bring in as many contributing factors as possible, including both biological and individual factors, to comprehensively explore and rank the risk factors for VL.

## 5. Conclusions

Due to the re-emergence of the disease in recent decades, VL remains a significant public health burden in central China. Based on the VL cases reported in central China from 2006 to 2019 obtained from the CDC, we ran the analysis on the spatiotemporal distribution characteristics of VL in the study area. The results show year-by-year increases in the number of VL cases. Additionally, most VL cases were distributed mainly in the eastern, southern, and central parts of Shaanxi Province and the southwestern and eastern parts of Shanxi Province. By combining VL case data with spatial predictors, we further explored the factors driving the spread of VL based on the GAM model, revealing significant correlations between environmental, meteorological, and socioeconomic factors and the prevalence of VL in central China. A significantly positive correlation between mean temperature and VL was observed here in the results. A complex nonlinear but generally negative association occurred between the variables of relative humidity and VL. This correlation shows a trend that first rises and then descends. The relationships between changes in forest and grass and VL were observed as U-shaped curves.

## Figures and Tables

**Table 1:** The spatial predictors adopted in this study.

Factor	Variables	Data Source
Environmental	Elevation	Shuttle Radar Topography Mission (SRTM)
	Change of forest	MODIS Land Cover Product (MCD12C1)
	Change of grassland	
Meteorological	Mean temperature	China Meteorological Data Service Centre (CMDC)
	Relative humidity	
	Precipitation	
Socioeconomic	Population density	Socioeconomic Data and Applications Centre (SEDAC)
	GDP	Global Change Research Data Publishing and Repository
	Urban accessibility	European Commission Joint Research Centre (ECJRC)

**Table 2:** Detailed information of endemic counties.

Province	City	County/District
Shanxi	Yangquan	Urban district (Pingding)
		Mining district (Yu)
		Suburban district
	Changzhi	Suburban district
		Wuxiang
	Linfen	Quwo
		Xiangfen
		Xiangning
		Daning
Shaanxi	Weinan	Linwei
		Huazhou
		Hancheng
	Yanan	Yichuan
	Yulin	Suide
		Qingjian
Henan	Anyang	Linzhou

**Table 3:** The temporal distribution of VL cases from 2006 to 2019 in central China.

Year	Shaanxi	Shanxi	Henan	Hebei	Total
	No. of Cases				
2006	2	1	0	0	3
2007	1	0	1	0	2
2008	3	1	0	0	4
2009	3	1	0	0	4
2010	2	2	0	0	4
2011	6	4	1	0	11
2012	8	2	0	1	11
2013	6	5	1	1	13
2014	14	4	0	2	20
2015	15	9	1	0	25
2016	13	10	4	2	29
2017	20	29	8	1	58
2018	31	41	7	2	81
2019	37	52	7	5	101
Total	161	161	30	14	366

**Table 4:** Statistical results of spatial predictors based on the GAM model.

Factor	Variables	Chi.sq/ Estimate	*P* Value
Environmental	Elevation	99.618 ^a^	0.000 ***
	Change of forest	13.747 ^a^	0.010 *
	Change of grassland	42.210 ^a^	0.000 ***
Meteorological	Mean temperature	72.594 ^a^	0.000 ***
	Relative humidity	87.193 ^a^	0.000 ***
	Precipitation	4.322 ^a^	0.262
Socioeconomic	Population	4.615 × 10^−5 b^	0.000 ***
	GDP	4.322 × 10^−5 b^	0.054 ^†^
	Urban accessibility	−1.195 × 10^−3 b^	0.038 *

Note: *** *P* < 0.001; * *P* < 0.05, ^†^ *P* < 0.1. ^a^ Chi.sq (chi-squared Test), ^b^ estimate.

## Data Availability

All relevant data are contained within the manuscript. The datasets used and/or analysed during the current study are available from the corresponding author on reasonable request.
